# Supplementary material for: MetaQTL: a package of new computational methods for the meta-analysis of QTL mapping experiments
Source: BMC Bioinformatics. 2007 Feb 8;8:49. doi: 10.1186/1471-2105-8-49 (PMC1808479; doi:10.1186/1471-2105-8-49)
Supplement: Additional File 7 — MetaQTL Package : jar file and tutorial. This Zip archive contains both the MetaQTL JAR file and the files of the tutorial. [file 1471-2105-8-49-S7.zip › org.inra.metaqtl/doc/org/metaqtl/Qtl.QTLTrait.html]

Qtl.QTLTrait


|  |  |  |
| --- | --- | --- |
| **Overview** \| **Package** \| **Class** \| **Use** \| **Tree** \| **Deprecated** \| **Index** \| **Help** | |  |
| **PREV CLASS**   **NEXT CLASS** | **FRAMES**    **NO FRAMES**     **All Classes** |  |
| SUMMARY: NESTED \| FIELD \| CONSTR \| METHOD | DETAIL: FIELD \| CONSTR \| METHOD |  |


---


## org.metaqtl Class Qtl.QTLTrait

```
java.lang.Object
  org.metaqtl.Qtl.QTLTrait
```

**All Implemented Interfaces:**: java.io.Serializable

**Enclosing class:**: Qtl

---

``` public class Qtl.QTLTrait extends java.lang.Object implements java.io.Serializable ```

This class stores the trait description of a QTL

**See Also:**: Serialized Form

---

| **Field Summary** | |
| --- | --- |
| `java.lang.String` | `group`             The name of the group to which the trait belongs. |
| `java.lang.String` | `name`             The name of the trait. |
| `java.lang.String` | `unit`             The unit of the trait. |


**Constructor Summary**

`Qtl.QTLTrait()`


| **Method Summary** | |
| --- | --- |
| `Qtl.QTLTrait` | `getCopy()` |

**Methods inherited from class java.lang.Object**

`clone, equals, finalize, getClass, hashCode, notify, notifyAll, toString, wait, wait, wait`

**Field Detail**

### name

```
public java.lang.String name
```

:   The name of the trait.

---


### unit

```
public java.lang.String unit
```

:   The unit of the trait.

---


### group

```
public java.lang.String group
```

:   The name of the group to which
    the trait belongs.


**Constructor Detail**

### Qtl.QTLTrait

```
public Qtl.QTLTrait()
```


**Method Detail**

### getCopy

```
public Qtl.QTLTrait getCopy()
```

:   **Returns:**


---


|  |  |  |
| --- | --- | --- |
| **Overview** \| **Package** \| **Class** \| **Use** \| **Tree** \| **Deprecated** \| **Index** \| **Help** | |  |
| **PREV CLASS**   **NEXT CLASS** | **FRAMES**    **NO FRAMES**     **All Classes** |  |
| SUMMARY: NESTED \| FIELD \| CONSTR \| METHOD | DETAIL: FIELD \| CONSTR \| METHOD |  |


---
